# A Novel Dental Sealant Containing Dimethylaminohexadecyl Methacrylate Suppresses the Cariogenic Pathogenicity of *Streptococcus mutans* Biofilms

**DOI:** 10.3390/ijms20143491

**Published:** 2019-07-16

**Authors:** Maria Salem Ibrahim, Ahmed S. Ibrahim, Abdulrahman A. Balhaddad, Michael D. Weir, Nancy J. Lin, Franklin R. Tay, Thomas W. Oates, Hockin H. K. Xu, Mary Anne S. Melo

**Affiliations:** 1 Ph.D Program in Dental Biomedical Sciences, University of Maryland School of Dentistry, Baltimore, MD 21201, USA; 2 Department of Preventive Dental Sciences, College of Dentistry, Imam Abdulrahman Bin Faisal University, Dammam 34212, Saudi Arabia; 3 Medical Microbiology Department, Health Monitoring Centers, Ministry of Health, Jeddah 21176, Saudi Arabia; 4 Department of Restorative Dental Sciences, College of Dentistry, Imam Abdulrahman Bin Faisal University, Dammam 34212, Saudi Arabia; 5 Department of Advanced Oral Sciences and Therapeutics, Division of Biomaterials and Tissue Engineering, University of Maryland School of Dentistry, Baltimore, MD 21201, USA; 6 Biosystems and Biomaterials Division, National Institute of Standards and Technology, Gaithersburg, MD 20899, USA; 7 Department of Endodontics, The Dental College of Georgia, Augusta University, Augusta, GA 30912, USA; 8 Center for Stem Cell Biology and Regenerative Medicine, University of Maryland School of Medicine, Baltimore, MD 21201, USA; 9 University of Maryland Marlene and Stewart Greenebaum Cancer Center, University of Maryland School of Medicine, Baltimore, MD 21201, USA; 10 Division of Operative Dentistry, Department of General Dentistry, University of Maryland School of Dentistry, Baltimore, MD 21201, USA

**Keywords:** antibacterial agents, biofilm, dental caries, sealant, quaternary ammonium compounds, *Streptococcus mutans*

## Abstract

Cariogenic oral biofilms are strongly linked to dental caries around dental sealants. Quaternary ammonium monomers copolymerized with dental resin systems have been increasingly explored for modulation of biofilm growth. Here, we investigated the effect of dimethylaminohexadecyl methacrylate (DMAHDM) on the cariogenic pathogenicity of *Streptococcus mutans* (*S. mutans*) biofilms. DMAHDM at 5 mass% was incorporated into a parental formulation containing 20 mass% nanoparticles of amorphous calcium phosphate (NACP). *S. mutans* biofilms were grown on the formulations, and biofilm inhibition and virulence properties were assessed. The tolerances to acid stress and hydrogen peroxide stress were also evaluated. Our findings suggest that incorporating 5% DMAHDM into 20% NACP-containing sealants (1) imparts a detrimental biological effect on *S. mutans* by reducing colony-forming unit counts, metabolic activity and exopolysaccharide synthesis; and (2) reduces overall acid production and tolerance to oxygen stress, two major virulence factors of this microorganism. These results provide a perspective on the value of integrating bioactive restorative materials with traditional caries management approaches in clinical practice. Contact-killing strategies via dental materials aiming to prevent or at least reduce high numbers of cariogenic bacteria may be a promising approach to decrease caries in patients at high risk.

## 1. Introduction

Dental caries is a biofilm-triggered oral disease with an international pandemic distribution, primarily affecting school-age children [1]. The World Health Organization reports that dental caries is the most common oral condition included in the Global Burden of Disease Study [2]. Carious lesions of permanent teeth (2.3 billion people) ranks as the most prevalent oral condition, while carious lesions on deciduous teeth (560 million children) ranks 12th [2]. The pits and fissures on children’s teeth present a topography with deep and narrow features, making the mechanical removal of plaque bacteria by brushing very challenging [3].

In efforts toward pediatric caries prevention, dental sealants were introduced for application on the occlusal surface. The sealants act as a physical barrier for microorganisms at the pits and fissures of the teeth. Evidence-based clinical recommendations, stated by the American Dental Association and the American Academy of Pediatric Dentistry, support sealants as a practical approach in preventing and arresting pit-and-fissure occlusal caries lesions [4]. Although sealants are a widely used preventive approach against occlusal carious lesions, the failure rate is high. Longitudinal data [5,6] show reasons for failure related to bacterial colonization underneath the sealed fissures with the progressive demineralization and development of cavitation.

Exploring the potential opportunities for new biotechnologies and biomaterials to improve dental outcomes requires an enhanced understanding of caries etiology and bacteria‒material interactions. A material that can detrimentally affect the oral bacteria on or near its surface is a promising area of dental material development. For instance, bioactive or bioinductive materials can help reduce cariogenic biofilm formation at the sealant‒tooth interface [7]. Likewise, contact-active dental materials that inhibit the growth of acid-producing bacteria can potentially disturb and even correct the localized dysbiosis (imbalance) of the oral microflora that may lead to caries around dental sealants [8,9]. Quaternary ammonium compounds are highly antimicrobial when applied in solution and also demonstrate antimicrobial activity when immobilized on a surface [10]. In the last decade, quaternary ammonium-based methacrylate monomers have been investigated to impart antibacterial surfaces to dental materials.

Recent works have demonstrated the application of dimethylaminohexadecyl methacrylate (DMAHDM) and amorphous calcium phosphate (NACP) as an antibacterial strategy for resin-based materials [8,11,12]. DMAHDM, an antibacterial monomer, acts primarily through direct contact killing [13]. It has been previously hypothesized that a positively charged DMAHDM structure may interact with negatively charged bacteria to lead to cell membrane leakiness and rupture [14,15]. Our previous report [16] has tuned sealant formulations to reach acceptable values for physical and mechanical properties using DMAHDM at 5 mass% and NACP at 20 mass%, respectively. However, the antibacterial performance of these formulations was not investigated.

The targeted cariogenic biofilm is a densely packed community of oral microbial cells with predominantly acid-tolerant, acid-producing bacteria surrounded by an exopolysaccharide (EPS)-rich matrix [13,17,18]. Within cariogenic dental plaque, *Streptococcus mutans* (*S. mutans*) has been strongly linked to cariogenic biofilm formation and carious lesion progression [19]. The main observable characteristics associated with *S. mutans* cariogenic pathogenicity include adhesion to tooth surfaces, synthesis of EPS via glucosyltransferases, biofilm formation, efficient use of sucrose to create an acidic environment, and aciduricity via acid tolerance response [13,20]. These virulence factors under a sucrose-rich environment contribute to the structural integrity and pathogenicity of the biofilm produced by *S. mutans* [17,18]. The remarkable ability of *S. mutans* to cope with significant and constant environmental variations, including changes in pH and oxygen tension, further leads it to be one of the primary cariogenic pathogens in this context [20].

The ability to reduce or modulate the virulence and viability of *S. mutans* biofilms using bioactive dental sealants could reduce caries associated with sealants. In the present study, we hypothesized that the formation and cariogenicity of *S. mutans* biofilms (acidogenicity, EPS production, aciduricity and tolerance to oxygen stress) would be reduced when the biofilms are in contact with sealant formulations containing 5% DMAHDM. Accordingly, this study aimed to investigate the effect of sealant formulations with 5% DMAHDM and 20% NACP on the virulence properties and viability of *S. mutans* biofilms.

## 2. Results

### 2.1. DMAHDM Reduced Biofilm Formation

**Figure 1:**
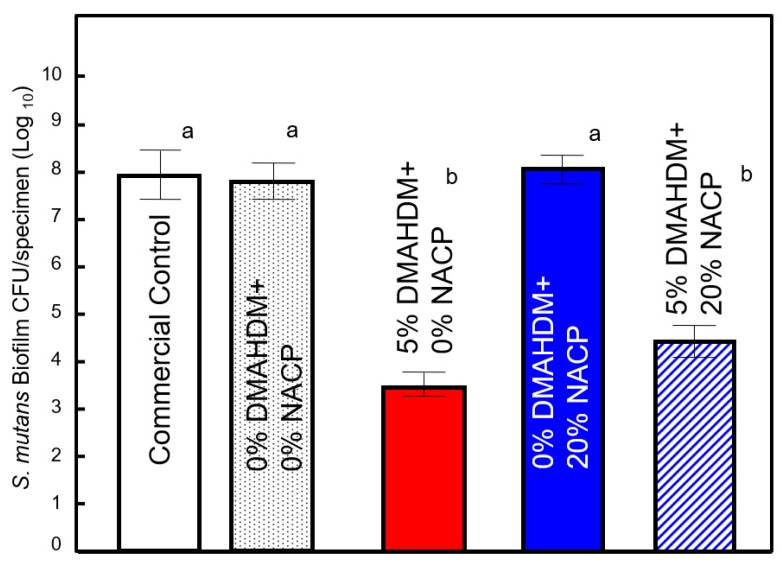
Colony-forming units per sealant specimen for *S. mutans* biofilms (mean ± SD of three independent experiments; *n* = 6 per group/experiment). Different lowercase letters denote a significant difference at a level of ∝ = 0.05 among groups.

Multiple properties of *S. mutans* were strongly reduced when the biofilms were cultured on the surfaces of sealants containing DMAHDM, relative to sealants without DMAHDM. Figure 1 shows the CFU counts for the 48 h *S. mutans* biofilms formed on the various sealants. The CFU values for groups containing 5% DMAHDM were significantly lower than those for control groups (commercial and experimental) and for groups containing 20% NACP (*p* < 0.05). A two-way ANOVA found a significant effect for 5% DMAHDM (F (1,58) = 5.453; *p* = 0.23) but no significant effect for 20% NACP (F (1,58) = 0.884; *p* = 0.351) or for the interaction between these factors (*p* = 0.88) on CFU counts.

**Figure 2:**
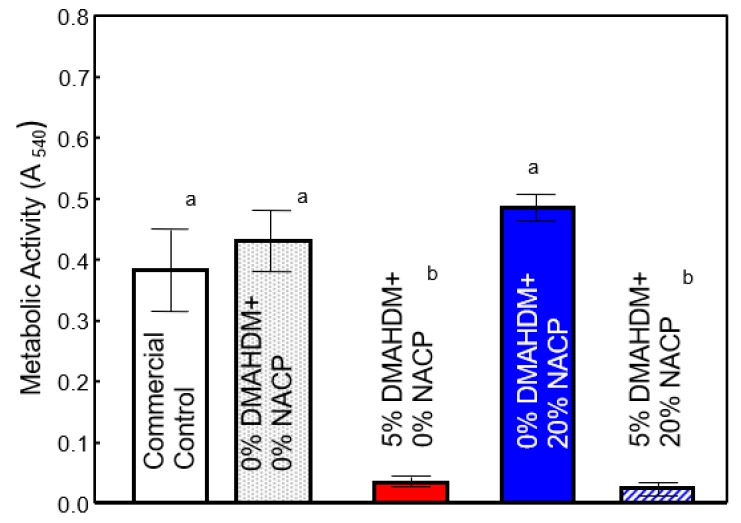
The metabolic activity of the 48 h *S. mutans* biofilms measured by MTT assay (mean ± SD of three independent experiments; *n* = 6 per group/experiment; non-normalized values). Different lowercase letters denote a significant difference at a level of ∝ = 0.05 among groups.

Figure 2 shows the results from the MTT assay reflecting active metabolizing (respiring) *S. mutans* cells in the biofilms adherent to the sealant specimens. For the two formulations containing 5% DMAHDM, the MTT values were lower in comparison to the control (*p* < 0.05), representing approximately 82% and 87% reduction in biofilm metabolic activity for 5% DMAHDM + 0% NACP and 5% DMAHDM + 20% NACP, respectively. There is no main effect for the presence of 20% NACP (F (1,64) = 0.784; *p* = 0.467).

### 2.2. DMAHDM Alone and in Combination with NACP Alters Acid and Oxygen Tolerance of S. mutans

**Figure 3:**
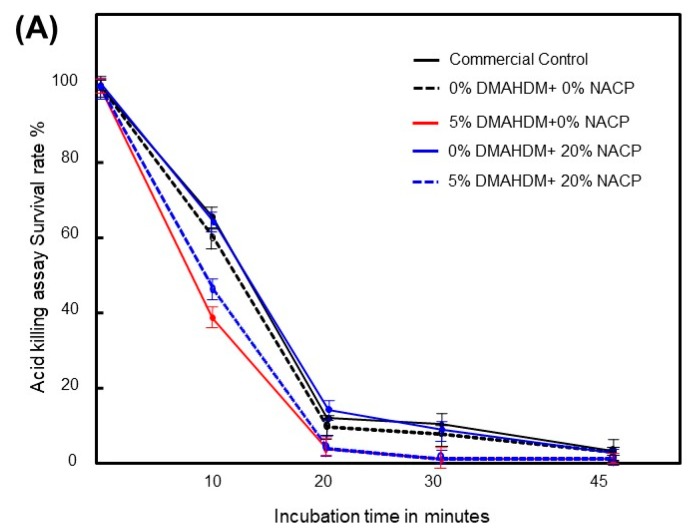

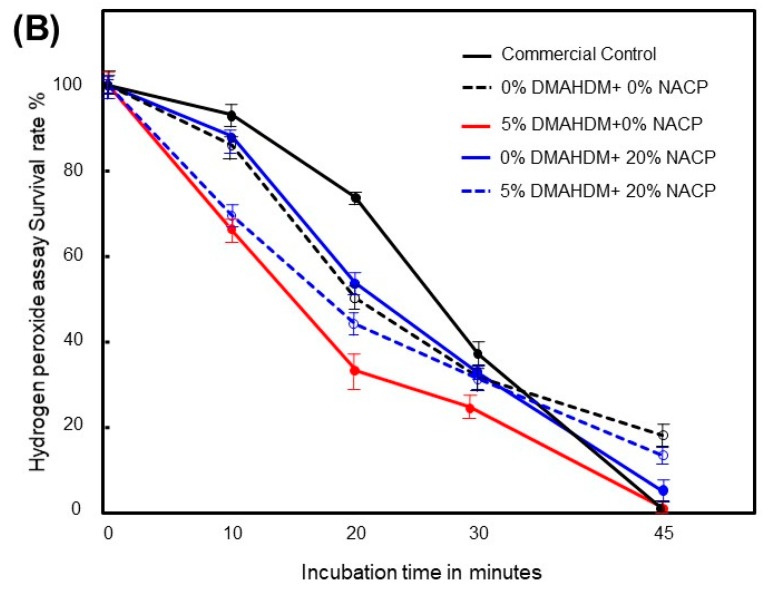
*S. mutans* biofilm survival expressed as the percent change in CFUs after exposure to (**A**) acid (pH 2.8) and (**B**) 0.2% H_2_O_2_ for up to 45 min (Mean ± SD of three independent experiments; *n* = 8 per group/experiment). Lines were drawn to improve readability.

Figure 3A uses the change in CFU (normalized to time zero) to represent the *S. mutans* biofilm survival rate after exposure to pH 2.8 for up to 45 min. *S. mutans* biofilms grown on sealant specimens containing 5% DMAHDM displayed lower survival at 10 min (38% and 44%, respectively) compared with the control and NACP only groups (60% and 65%, respectively). This difference was not as pronounced at later time points.

For the hydrogen peroxide killing assay, Figure 3B depicts the survival rate of *S. mutans* biofilms exposed to an H_2_O_2_ concentration of 0.2% for 45 min. *S. mutans* biofilms formed on sealant specimens containing 5% DMAHDM exhibited lower survival than the control sealant specimens at all timepoints except at 30 min and 45 min.

### 2.3. Attenuation of Exopolysaccharide Produced by S. mutans

**Figure 4:**
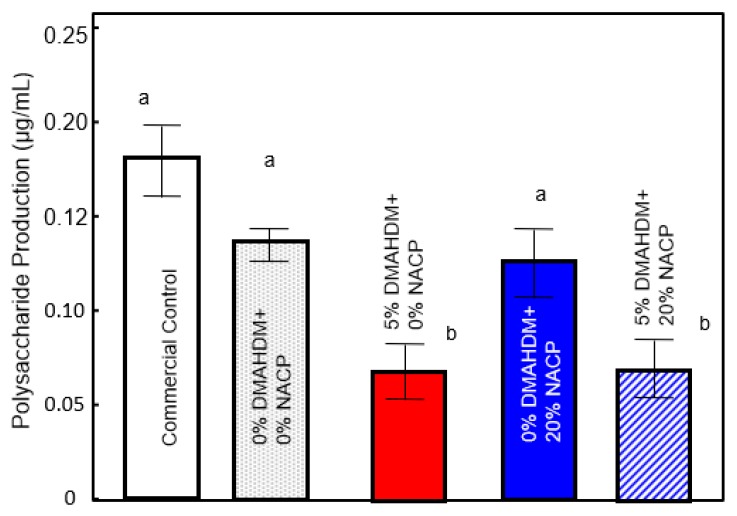
The polysaccharide production of 48 h *S. mutans* biofilms expressed as µg/mL (mean ± SD of three independent experiments; *n* = 6 per group/experimental). Different lowercase letters denote a significant difference at a level of ∝ = 0.05 among groups.

Measurements of EPS levels in the biofilms are plotted in Figure 4. Biofilms on the control sealant specimens had high exopolysaccharide production. Adding only 20% NACP to the formulation did not change the production of the polysaccharide (F_1,23_ = 0.626, *P* = 0.436) substantially from the controls. Incorporation of DMAHDM significantly decreased the water-insoluble polysaccharide synthesis from *S. mutans* (*p* < 0.05). There was no significant interaction between DMAHDM and NACP content for polysaccharide production.

**Figure 5:**
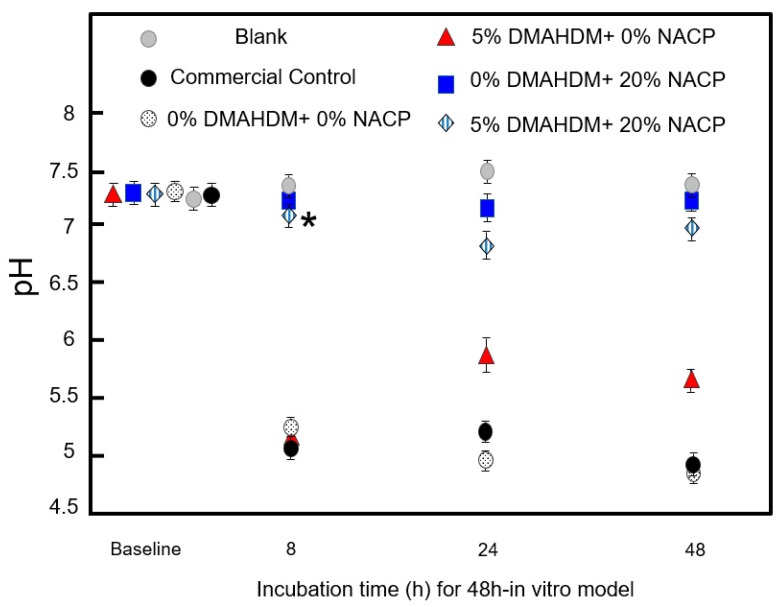
pH measurements (mean ± SD; *n* = 3) of growth medium from *S. mutans* biofilms grown on the various sealant specimens for 0 h, 8 h, 24 h, and 48 h. The blank represents growth medium incubated alongside tested groups. The asterisk represents a significant difference at a level of ∝ = 0.05 for 0% DMAHDM + 20% NACP and 5% DMAHDM + 20% NACP.

Figure 5 plots the pH of the culture medium during biofilm formation. Biofilms in commercial and experimental control groups had the highest acid production, leading to a drop in pH sustained over 48 h. The addition of 20% NACP supported a near neutral pH over the 48-hour time course (*p* < 0.05). The addition of 5% DMAHDM had a minimal effect when NACP was present, but without NACP the initial pH drop within the first 8 h matched that of the controls. At 24 h and 48 h, however, the pH in the 5% DMAHDM only-sealant specimens increased relative to the controls, with pH values above 5.5.

**Figure 6:**
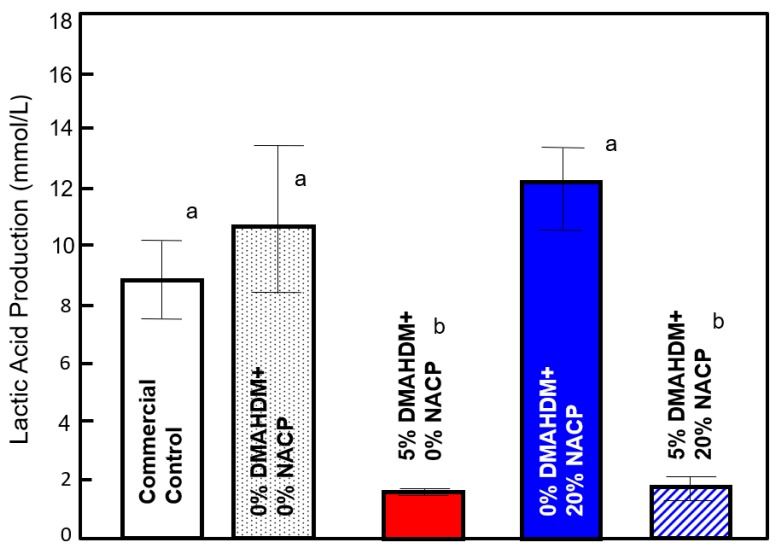
Lactic acid production of the 48 h *S. mutans* biofilms expressed as mmol/L (mean ± SD of three independent experiments; *n* = 6 per group/experimental). Different lowercase letters denote a significant difference at a level of ∝ = 0.05 among groups.

The lactic acid production of biofilms on the sealants is presented in Figure 6. Biofilms on the control groups and the group containing 20% NACP only produced the most acid (*p* < 0.05), and 20% NACP had no significant effect on lactic acid production. Analysis of the lactic acid concentration indicates a significant effect of DMAHDM incorporation (F_1,40_ = 4.611, *P* = 0.027) on the biofilm lactic acid production. Low lactic acid concentrations at levels approximately 10% of those on the control groups were observed. There was no interaction for lactic acid production between DMAHDM content and NACP content.

**Figure 7:**
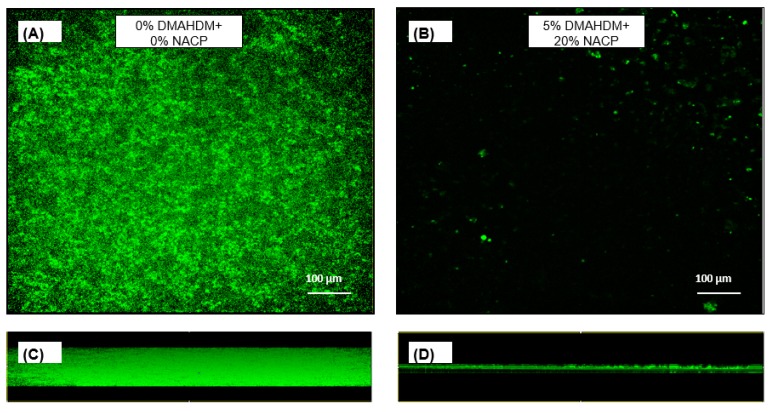
Scanning confocal laser microscope images of *S. mutans* biofilms formed on 0% DMAHDM + 0% NACP (control) and 5% DMAHDM + 20% NACP. (**A**,**B**) Random, representative images in the x‒y plane (top view); (**C**,**D**) random, representative images in the x‒z plane (side view).

Imaging of green fluorescent protein (GFP)-expressing *S. mutans* was used to visualize the biofilm structures. The 48 h *S. mutans* biofilms formed on the 5% DMAHDM + 20% NACP sealant specimens (Figure 7A) were qualitatively reduced in visible biomass as compared to biofilms that grew on the control sealant specimens (Figure 7B). The biofilms on the control sealants (Figure 7C) appeared denser and thicker in comparison to biofilms on the DMAHDM + NACP sealant (Figure 7D).

## 3. Discussion

In this study, we explored the influence of an antibacterial contact killing approach for sealant formulations on virulence factors of *S. mutans*. The antibacterial strategy of incorporating 5% DMAHDM into a parental formulation containing 20% NACP resulted in an overall significant reduction of acid-producing biofilms. The data obtained from the CFU (Figure 1) and metabolic activity (Figure 2) assays showed that 5% DMAHDM, alone or combined with 20% NACP, can remarkably inhibit the activity of *S. mutans* biofilms, surpassing the clinically relevant greater-than-3-log (99.9%) reduction [21]. These results are consistent with previous reports for oral biofilms exposed to dental bonding formulations containing DMAHDM [22,23].

Subsequently, we examined whether the in vitro antibiofilm activity of DMAHDM would translate into changes in the ability of *S. mutans* to survive and resist high acid and oxygen stresses. *S. mutans* needs to present aciduricity to avoid falling victim to its own acidogenic metabolism. However, biofilms grown on DMAHDM-containing sealants were generally less tolerant of acidic and oxidative stresses (Figure 4). Upon exposure to acid, *S. mutans* promotes many changes at the transcriptional and physiological levels to respond to the threat of acid damage to sensitive and critical cytoplasmic molecules, such as metabolic organelles and DNA [24,25]. *S. mutans* protects itself from the damaging effects of acids through the maintenance of a cytoplasm that is more alkaline relative to the extracellular space [26]. This alkaline environment is mainly reached via plasma membrane mechanisms such as F1F0 ATPase for proton extrusion and changes in unsaturated fatty acid percentage [24]. The harmful interactions between DMAHDM and the *S. mutans* membrane can hypothetically damage a cell’s ability to maintain the intracellular alkaline environment. Similarly, *S. mutans* makes changes to its membrane composition to limit the formation of hydrogen peroxide, promote its safe elimination, and repair damage [27]. If the membrane is dysfunctional, such as via perturbation by quaternary ammonium compounds like DMAHDM, the cell may have a reduced ability to survive in H_2_O_2_ environments, as seen here.

*S. mutans* relies on exopolysaccharide matrix synthesis to help it attach firmly to tooth surfaces. *S. mutans’* ability to synthesize exopolysaccharide is essential for a biofilm’s structural stability and may limit antibacterial strategies [28]. According to the results of the EPS assay (Figure 5), the DMAHDM monomer lowers the level of extracellular polysaccharide production, which may make these biofilms less stable and may potentially be beneficial for the non-cariogenic microbial community. The representative confocal microscopy images (Figure 7) qualitatively support the results from the bioassays by showing reduced biofilm growth on the DMAHDM materials. The images suggest a reduced ability of *S. mutans* to form a dense biofilm architecture.

The presence of NACP alone (without DMAHDM) did not affect biofilm parameters including CFUs, metabolic activity, or polysaccharide production; however, an effect of NACP was observed on the pH values for the 48 h biofilm model where the pH stayed neutral for all time points tested when NACP was included in the formulation. NACP can affect pH through the release of supersaturating levels of calcium and phosphate ions. NACP is expected to enhance the remineralizing capacity relative to larger amorphous calcium phosphate particles due to the higher surface area-to-volume ratio of NACP. For instance, NACP has a relatively high specific surface area of 17.76 m^2^/g, compared to about 0.5 m^2^/g of traditional CaP particles [29]. The higher proportion of exposed surface area for NACP requires lower filler levels (by mass) to reach the same surface area and therefore expected outcome as compared to amorphous calcium phosphate on the micro scale. Previous studies showed that nanocomposites with NACP filler fraction of 20 mass% were able to reduce demineralization [30,31]. The importance of the calcium reservoir relies on the fact that Ca^2+^, being one of the ions of hydroxyapatite, may reduce the driving force for tooth demineralization that occurs during a pH drop in dental biofilm. During demineralization, calcium release precedes phosphate release from enamel, dentin, and cementum, and calcium-deficient carbonated hydroxyapatite comprises the major substitution activity that takes place [32]. The presence of carbonates and other ionic substitutions significantly disrupts the crystal lattice in hydroxyapatite, weakening the hydroxyapatite and increasing its susceptibility to acid attack and solubility. Therefore, using high-calcium release materials to suppress the demineralization process could be beneficial.

Collectively, our data support the hypothesis that sealants with DMAHDM are able to disrupt *S. mutans* biofilm formation, thereby reducing their virulence potential. This outcome helps open the door for an important pathway to suppress dental caries via biofilm modulation and not only total biofilm eradication. A previous study conducted to investigate the antibacterial effect of DMAHDM on multispecies biofilms revealed that biofilms had a reduced relative amount of *S. mutans* when grown in contact with 3% DMAHDM as compared to the control, suggesting that DMAHDM may also be able to modulate the biofilm species composition toward a non-cariogenic tendency [33]. Shifting the biofilm composition from a cariogenic/acidogenic phenotype to a less virulent or even non-cariogenic biofilm is essential. However, the *S. mutans*/quaternary ammonium interactions involve various complex mechanisms where specific mechanisms and pathways are not clear and are still under debate [10,34]. While this study focused on the ability of sealant formulations with 5% DMAHDM and 20% NACP to alter the virulence-related traits of *S. mutans*, further research on the underlying mechanisms of bacterial/quaternary ammonium interactions is needed to optimize materials and achieve desired outcomes of improved oral health.

## 4. Materials and Methods

### 4.1. Development of Dental Resin Sealant Formulations

The parental resin matrix [16] consisted of (% by mass): 44.5% of pyromellitic glycerol dimethacrylate (PMGDM) (Hampford, Stratford, CT, USA); 39.5% of ethoxylated bisphenol a dimethacrylate (EBPADMA) (Sigma-Aldrich, St. Louis, MO, USA); 10% of 2-hydroxyethyl methacrylate (HEMA) (Esstech, Essington, PA, USA); 5% of bisphenol a glycidyl dimethacrylate (Esstech), and 1% of phenyl bis (2,4,6-trimethyl benzoyl)-phosphine oxide (BAPO) (Sigma-Aldrich) as a photo-initiator. For some formulations, DMAHDM at 5 mass% was added. The mass ratio of resin to filler was 1:1, where the filler fraction consisted of silanized barium boroaluminosilicate glass particles (average size of 1.4 µm (Caulk/Dentsply, Milford, DE, USA)) with or without the addition of NACP. DMAHDM and NACP were synthesized according to previous studies [16,22].

The tested dental sealant formulations were prepared as follows (% by mass):

1. Commercial High-viscosity Sealant/Flowable Composite control termed “Commercial Control” (Virtuoso, DenMat, Lompoc, CA, USA).
2. Experimental Control Sealant termed “0% DMAHDM + 0% NACP; Experimental Control” (50% PEHB + 0% DMAHDM + 50% Glass + 0% NACP).
3. Experimental Sealant termed “5% DMAHDM + 0% NACP” (45% PEHB + 5% DMAHDM + 50% Glass + 0% NACP).
4. Experimental Sealant termed “0% DMAHDM + 20% NACP” (50% PEHB + 0% DMAHDM + 30% Glass + 20% NACP).
5. Experimental Sealant termed “5% DMAHDM + 20% NACP” (45% PEHB + 5% DMAHDM + 30% Glass + 20% NACP).

### 4.2. Sample Preparation

Sealant specimens (diameter = 9 mm; thickness = 2 mm) were prepared using polytetrafluoroethylene molds covered with Mylar strips and glass slides [35]. Each sealant specimen was light-cured (1200 mW/cm^2^; 60 s; Labolight DUO, GC America, Alsip, IL, USA) on each side. Specimens were sterilized using ethylene oxide (24-h cycle; Andersen Sterilizers, Inc., Haw River, NC, USA) and allowed to de-gas for at least seven days after sterilization.

### 4.3. S. mutans Biofilm Formation

An *S. mutans* biofilm model was used [36] with modifications. *S. mutans* UA159 from the American Type Culture Collection (ATCC, Manassas, VA, USA) was cultured overnight (≈18 h) in brain heart infusion (BHI) broth (Sigma-Aldrich) at 37 °C and 5% CO_2_ (by volume). This *S. mutans* culture was adjusted to an optical density at 600 nm (OD_600_) of 0.9 (≈1 × 10^8^ CFU/mL according to growth curves monitored by spectrophotometry) and diluted in biofilm medium containing BHI broth supplemented with 2% (by mass) sucrose to prepare the inoculum.

The inoculum (1.5 mL) was added to wells of a 24-well culture plate containing the sterilized sealant specimens, and the well plates were incubated in an anaerobic chamber (37 °C; gas composition by volume: 10% H_2_, 5% CO_2_, 85% N_2;_ Whitley Workstation DG250; Microbiology International, Frederick, MD, USA) for 48 h without agitation to form a more established biofilm. The growth medium was changed at 8 h and 24 h. Control groups included wells with 1.5 mL of inoculum without sealants (Control inoculum) and wells with 1.5 mL of BHI + 2% sucrose without bacteria (Control BHI). Sealant specimens with the 48-hour biofilms were characterized as described in Section 4.4, Section 4.5, Section 4.6, Section 4.7, Section 4.8, Section 4.9 and Section 4.10.

### 4.4. Colony-Forming Unit Counts

After the sealant specimens (*n* = 6) had been incubated for 48 h, each sealant specimen with the attached biofilm was washed in 1 mL of phosphate-buffered saline (PBS), transported to a vial containing 2 mL of PBS, sonicated for 5 min (Branson 3510-DTH Ultrasonic Cleaner), and vortexed (5 s; maximum speed) to harvest the biofilm [37]. The resultant bacterial suspensions were serially diluted 10^1^- through 10^6^-fold, drop-plated onto BHI agar plates, and anaerobically incubated at 37 °C. After 48 h, the number of colony-forming units (CFU) was determined [37]. The results were calculated based on the number of CFU and the dilution factor; log_10_ transformed data are expressed as CFU/specimen.

### 4.5. Metabolic Activity

Metabolic activity was assessed via MTT (3-[4,5-dimethylthiazol-2-yl]-2,5-diphenyltetrazolium bromide) assay [37]. Briefly, each sealant specimen (*n* = 6) was washed in 1 mL of PBS and transported to a new 24-well plate containing 1 mL of tetrazolium dye (0.5 mg/mL) in PBS. Then, sealant specimens were incubated anaerobically for 1 h and transferred to a new 24-well plate. To solubilize the formazan crystals, 1 mL of dimethyl sulfoxide (DMSO) was added to each well, and the plate was incubated for 20 min at 37 °C. After that, 200 μL of the DMSO solution was collected from each specimen to measure the absorbance at 540 nm [37] (SpectraMax, Molecular Devices LLC, San Jose, CA, USA). The OD_540_ of DMSO defines the blanks for the spectrophotometric method. Values were not normalized prior to analysis.

### 4.6. Acid Stress and Oxygen Stress Tolerance

The differences in acid tolerance of *S. mutans* UA159 biofilms on the various formulations were evaluated using the method described by Kim et al. [38]. After 48 h of incubation, sealant specimens (*n* = 8) were washed once with 0.1 mol/L glycine (pH 7.0) and switched to 0.1 mol/L glycine at pH 2.8. Wells with biofilms but not treated with glycine served as controls. Specimens were incubated at room temperature, and 100 μL aliquots were collected at 0 min, 10 min, 20 min, 30 min, and 45 min. CFU was determined per Section 4.4.

The differences in oxidative stress tolerance of *S. mutans* UA159 biofilms were evaluated via hydrogen peroxide killing assay (*n* = 8) [38]. Sealant specimens were washed once with 0.1 mol/L glycine (pH 7.0) and placed in 5 mL of the same buffer. After that, hydrogen peroxide was added for a final concentration of 0.2%, and 100-μL aliquots were collected at 0 min, 10 min, 20 min, 30 min, and 45 min after the addition of hydrogen peroxide. Ten microliters of catalase (5 mg/mL) were added to each aliquot to inactivate the hydrogen peroxide immediately after aliquot collection. CFU was determined per Section 4.4.

### 4.7. Polysaccharide Production

The water-insoluble polysaccharide production in the 48 h *S. mutans* biofilms was measured using a phenol-sulfuric acid method [9] with modifications. Each sealant specimen (*n* = 6) was rinsed with 2 mL of PBS, transferred to a vial containing 1 mL of PBS, sonicated (Branson 3510-DTH Ultrasonic Cleaner) for 5 min, and vortexed (5 s; maximum speed) to harvest the biofilm. Centrifugation (10,000× *g* (~10,000 r.c.f.) at 4 °C for 5 min) yielded a precipitate that was rinsed with PBS and resuspended in 1 mL of de-ionized water. Then, 1 mL of 6% (by volume) phenol solution was added to the vial, followed by 5 mL of 95–97% (by volume) sulfuric acid. After 30 min of incubation at 23 °C, 100 μL were transferred to a 96-well plate, and absorbance was measured at OD_490_ using a spectrophotometer. Standard glucose concentrations were used to convert OD readings to polysaccharide concentrations.

### 4.8. Acid-Neutralizing Activity

The pH of the biofilm growth medium (*n* = 3) was measured at 0 h, 8 h, 24 h, and 48 h using a digital pH meter (accuracy ± 5%; Accumet XL25, Thermo Fisher Scientific, Waltham, MA, USA). During the experiments, the pH meter was calibrated at regular intervals using commercial standard buffer solutions of pH 4, pH 7, and pH 10 at room temperature.

### 4.9. Lactic Acid Production

Lactate concentrations were determined using an enzymatic (lactate dehydrogenase) method [33]. After the sealant specimens had incubated for 48 h, each specimen (*n* = 6) with the attached biofilm was washed in 1.5 mL of cysteine peptone water (by mass: yeast extract 0.5%, peptone 0.1%, sodium chloride 0.85%, cysteine 0.005%), transferred to a new plate containing 1.5 mL of buffered peptone water (BPW) with 0.2% (by mass) sucrose, and incubated for 3 h at 37 °C in 5% CO_2_ [37]. The lactic acid concentrations in the BPW solution were then measured based on the absorbance at 340 nm using a spectrophotometer. A standard curve was prepared using lactic acid standard solution (46937; Sigma) [39].

### 4.10. Confocal Laser Scanning Microscopy

Confocal laser scanning microscopy (CLSM) was performed for qualitative visualization of fully-hydrated, living cultures for two groups of sealants: the experimental control and 5% DMAHDM + 20% NACP. The qualitative approach was chosen to support the microbiological assay results. Biofilms were grown as described apart from the bacterial strain. For imaging, a green fluorescent protein (*gfp*)-expressing strain of *S. mutans* UA159 (*gfp*-UA159) was used in the biofilm model. *Gfp*-UA159 cells continually exhibit green fluorescence throughout growth.

The fluorescence of *gfp*-UA159 48 h biofilms was captured with a Yokogawa Spinning Disk Field Scanning Confocal System (CSU-W1) (Nikon Instruments Inc. Melville, NY, USA) on a Nikon Ti2 inverted microscope (Nikon Instruments Inc. Melville, NY, USA) using a 20× (numerical aperture, 1.00) water immersion objective. Biofilms were imaged four times on two sealant specimens. Biofilm slices in the x‒y and x‒z planes were collected at random locations on the biofilms.

### 4.11. Statistical Analysis

All experiments were performed with six to eight repetitions (sealant specimens) in each of three independent experiments. The average of the three experiments per specimen was considered as a statistical unit. The data satisfied the assumption of the equality of variances and normal distribution of errors. Factors included DMAHDM incorporation at two levels (0% and 5%) and NACP incorporation at two levels (0% and 20% NACP). Interactions between these two factors were also considered. To assess the *S. mutans* biofilm biological response, the following dependent variables were analyzed: Log CFU/specimen, metabolic activity, lactic acid production, and exopolysaccharide synthesis. The means and standard deviations (which serve as an estimate of the measurement uncertainty) for the dependable variables were analyzed by two-way ANOVA. Statistical significance was pre-set at α = 5%. All the statistical analyses were performed by SPSS statistics software (IBM version 26, Armonk, NY, USA).

### 4.12. Disclaimer

Certain commercial equipment, instruments, and materials are identified in this paper in order to specify the experimental procedure. Such identification does not imply recommendation or endorsement by the National Institute of Standards and Technology (NIST), nor does it imply that the material or equipment identified is necessarily the best available for the purpose.

## 5. Conclusions

*S. mutans* relies on its virulence-related traits to change oral plaque biofilms and promote the development and increase the severity of carious lesions in children. This study investigated the antibacterial strategy of incorporating 5% DMAHDM into dental sealants containing 20% NACP to reduce virulence and viability of *S. mutans* biofilms. Our findings suggest that sealants with 5% DMAHDM were able to impart a detrimental biological effect on *S. mutans* biofilms by significantly reducing the biofilm formation, metabolic activity, and EPS production. The antibacterial sealants were also able to reduce the aciduricity and the tolerance to oxygen stress. This less virulent phenotype is expected to reflect on the clinical pathogenicity of *S. mutans* and to play a key role in improving the longevity and integrity of dental sealants. Contact-killing strategies via dental materials aiming to prevent or modulate pathogenic biofilms are a promising approach in patients at high risk of caries.
